# A Systematic Review of Athletes’ and Coaches’ Nutrition Knowledge and Reflections on the Quality of Current Nutrition Knowledge Measures

**DOI:** 10.3390/nu8090570

**Published:** 2016-09-16

**Authors:** Gina L. Trakman, Adrienne Forsyth, Brooke L. Devlin, Regina Belski

**Affiliations:** 1 School of Allied Health, La Trobe University, Melbourne 3086, Australia; A.forsyth@latrobe.edu.au (A.F.); R.Belski@latrobe.edu.au (R.B.); 2 Mary MacKillop Institute for Health Research, Australian Catholic University, Melbourne 3000, Australia; Brooke.Devlin@acu.edu.au

**Keywords:** nutritional knowledge, dietary knowledge, athlete, coach, sport, questionnaire, survey, measure, valid, sports nutrition

## Abstract

Context: Nutrition knowledge can influence dietary choices and impact on athletic performance. Valid and reliable measures are needed to assess the nutrition knowledge of athletes and coaches. Objectives: (1) To systematically review the published literature on nutrition knowledge of adult athletes and coaches and (2) to assess the quality of measures used to assess nutrition knowledge. Data Sources: MEDLINE, CINAHL, SPORTDiscuss, Web of Science, and SCOPUS. Study Selection: 36 studies that provided a quantitative measure of nutrition knowledge and described the measurement tool that was used were included. Data extraction: Participant description, questionnaire description, results (mean correct and responses to individual items), study quality, and questionnaire quality. Data synthesis: All studies were of neutral quality. Tools used to measure knowledge did not consider health literacy, were outdated with regards to consensus recommendations, and lacked appropriate and adequate validation. The current status of nutrition knowledge in athletes and coaches is difficult to ascertain. Gaps in knowledge also remain unclear, but it is likely that energy density, the need for supplementation, and the role of protein are frequently misunderstood. Conclusions: Previous reports of nutrition knowledge need to be interpreted with caution. A new, universal, up-to-date, validated measure of general and sports nutrition knowledge is required to allow for assessment of nutrition knowledge.

## 1. Introduction

A carefully planned nutrition program has significant positive effects on athletic performance [1,2,3]. There has recently been an increase in internationally endorsed dietary guidelines for athletes, reflected by the publication of several consensus statements on optimal intake and timing of food, fluid, and supplements [4,5]. Despite this, research indicates that many athletes have sub-optimal dietary intakes [6,7], which may be due to lack of time, finances, cooking skills, and access to cooking equipment when attempting to select and prepare appropriate meals and snacks [8]. Food choices may also be driven by factors such as cultural background, taste preferences, appetite, attitude towards nutrition, and nutrition knowledge [8,9,10].

Nutrition knowledge is one of the few modifiable determinants of dietary behaviors. Sports dietitians often center their dietary interventions on nutrition education to improve awareness of and compliance with expert dietary guidelines [10,11]. Nutrition education programs are rarely evaluated. There are a number of cross-sectional studies reporting on the nutrition knowledge of both athletes and coaches [12,13,14]. In a 2011 systematic review of the nutrition knowledge of recreational and elite athletes, scores across various nutrition knowledge questionnaires assessing general and sports specific nutrition were mediocre, with mean scores of approximately 45%–65% [7]. There appeared to be a weak, positive correlation between nutrition knowledge and good quality dietary intake. The review concluded that in order to confirm the nutrition knowledge of athletes, and the relationship between nutrition knowledge and dietary intake, further high-quality research was required [7]. A 2014 review on the relationship between nutrition knowledge and dietary intake in adults also suggested that while the relationship between nutrition knowledge and dietary behavior appears to be moderate at best, results may be affected by the quality of measures used to assess knowledge [6]. Several studies assessing nutrition knowledge in athletes, not included in either of the aforementioned reviews, have been published in recent years [12,15,16,17,18,19,20,21,22,23].

Despite researchers having raised concerns regarding the validity of current nutrition knowledge measures [6,7,22], a detailed review of their limitations has not been undertaken to date. It is important to consider the comprehensiveness of the tools used. That is, the extent to which they have assessed all the relevant topics of nutrition knowledge, such as knowledge of macronutrients, micronutrients, supplementation, and hydration. In nutrition knowledge measures, questions on each of these topics are often grouped together and referred to as nutrition “sub-sections”. Previous reviews have identified concerns with drawing comparison between studies due to the heterogeneity of measures used; however, analysis of related nutrition sub-sections and responses to congruent questions across studies has not been performed. Several reports [9,11] and cross-sectional studies in elite Australian athletes and American College athletes have established that coaches are often a key source of nutrition information for athletes [16,24,25] but there has not been a systematic review of their nutrition knowledge. 

Considering the importance of nutrition knowledge as a modifiable determinant of dietary behavior, the aims of the present review are to determine whether:

1. Athletes (aged 17 years and over) and coaches of adult athletes are aware of expert nutrition recommendations
2. There are gaps in particular topics (nutrition sub-sections) of nutrition knowledge
3. The quality (validity, reliability, and comprehensiveness) of measures that have been used to assess nutrition knowledge is acceptable.

## 2. Methods

### 2.1. Protocol and Registration

Methods for the review were in accordance with PRISMA guidelines and were registered with PROSEPERO [26].

### 2.2. Search Terms

A systematic search using the strategy nutrition knowledge or diet knowledge and athlete or sports people or sportsman and questionnaire or tool or measure or survey and valid or reliable, was conducted by one researcher (GT) from the earliest record until November 2015. A second search using the terms nutrition knowledge or diet knowledge and coach or questionnaire or tool or measure or survey was also conducted. Searched databases included MEDLINE, CINAHL, SPORTDiscuss, Web of Science, and SCOPUS. To ensure all related texts were captured, the reference lists of included articles were hand-searched.

### 2.3. Eligibility Criteria

Original research (cross-sectional, observational, randomized controlled trials) conducted in adult athletes (17 years and older) or coaches/athletic trainers of adult athletes, and published in peer-reviewed journals were included for review. Abstracts, conference posters, reviews, and unpublished theses were excluded. Athletes were defined as individuals involved in training and playing competitive sport. All ‘levels’ of athletic competition, for example, recreational, college, national, and international were accepted. Only English language studies were included. Studies needed to report an aspect of nutrition knowledge (general, overall sports, or specific sports nutrition e.g., hydration) using a measure that produced a numerical score. Studies that provided qualitative data only, or stated how many participants answered questions correctly/incorrectly, but failed to report overall quantitative results were excluded. The questionnaires could be in any format including self-administered, researcher-administered, online, or handwritten. To be included, studies also needed to provide a description of the tool used to assess knowledge including number of items, content, and question response categories (Table 1). 

### 2.4. Selection Process

**Figure 1:**
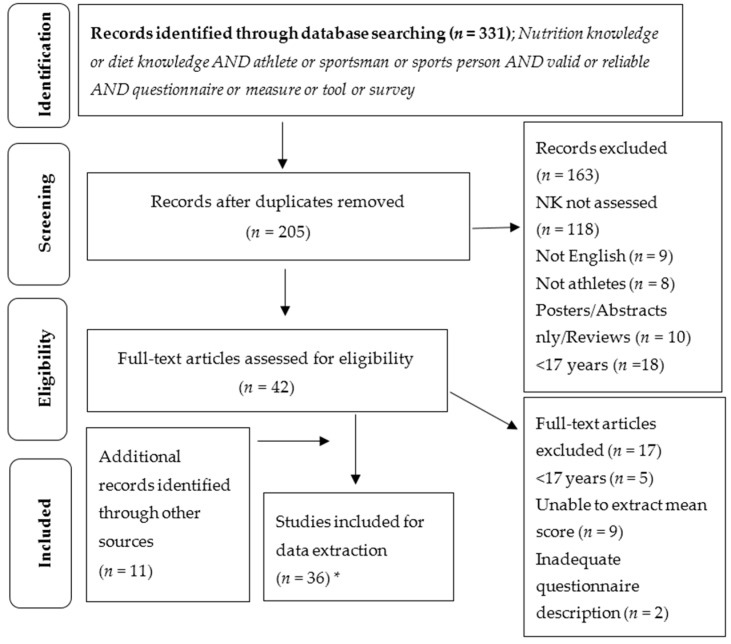
Flowchart of review process. * Secondary search using the term coach did not yield any additional relevant articles. NK = nutrition knowledge.

Duplicate and irrelevant articles were excluded on the basis of abstract and title by two authors (GT and AF). Articles deemed eligible for full-text review were retrieved and screened against the inclusion criteria by two authors (GT and AF) (Figure 1).

### 2.5. Data Extraction and Tool Quality

Data from eligible studies were extracted by one author (GT). Information retrieved included: country of study, participant description (age, gender, sport played/coached, athletic level), questionnaire description (item generation, number of questions, question-response format), and results (mean nutrition knowledge scores, as well as nutrition sub-sections where participants scored above and below the study’s overall mean). All scores were converted into percentage correct for consistency. Athletic level was based on descriptions provided in the paper; if athletic level was not adequately described, judgments on athletic level were based on other available information such as participant recruitment. Where reported, responses to individual items were also extracted then collated and summarized based on congruent themes. If questionnaires were not available, authors were contacted and permission to review a copy of the tool that was used was requested.

Detailed data on the quality of the measures reported in the studies reviewed were recorded and used to calculate two separate quality scores: one for validity and reliability and another for questionnaire comprehensiveness. The validity and reliability score was based on a set of guidelines developed by Parmenter and Wardle [27]. Their recommendations are based upon psychometric validation techniques within the classical test theory (CTT) framework and are in line with leaders in the field of scale development, such as Kline [28] and Nunnally [29]. They outline several methods for the development and evaluation of questionnaires, including: item analysis (item difficulty/item discrimination); homogeneity/”internal consistency” assessed using Cronbach’s alpha; face validity assessed using a cohort similar to the target audience; content validity assessed using a panel of experts; construct validity assessed using known-group comparisons; and test–retest reliability using Pearson’s correlation. In accordance with these guidelines, a validity score out of six was given. The decision was made to assess face validity, as, although it is similar to content validity, it utilizes different focus groups (target audience, not experts) and has different aims (ensuring readability/tool assesses what it intends to, not ensuring the entire content of the domain is covered). Scales developed under CTT apply only to the group of people who took the test; therefore, it is necessary to re-run internal consistency calculations for new samples [30]. Accordingly, in instances where an existing measure or modified version of an existing tool was used, a point was not awarded for internal consistency unless Cronbach’s alpha was reassessed. If this test had been performed in the original sample, a partial point was given (denoted by P). If a tool had been modified from a previous tool, or was a composite of various previous questionnaires, validation points were not awarded unless the new version had undergone psychometric testing. 

For the comprehensiveness score, a point was awarded for each of the following nutrition sub-sections covered: general nutrition knowledge, carbohydrates, proteins, fats, micronutrients, hydration, pre-exercise nutrition, nutrition during competition, recovery nutrition, supplementation, and alcohol. A maximum of 11 points could be awarded. Decisions on whether a questionnaire included adequate coverage on each topic to be included as a nutrition sub-section were made by one author (GT), based on a combination of review of the actual tool (when available) and the description of the measure provided in the article.

### 2.6. Study Quality

The methodological quality of studies was assessed by two reviewers (GT and AF), using the “Academy of Nutrition and Dietetics” “Quality Criteria Checklist for Primary Research” [31]. Disagreements were resolved by a third reviewer (BD). The checklist rates studies as positive, neutral, or negative (poor) on 10 criteria. The criteria addressing study group comparisons (3), methods for handling withdrawals (4), use of blinding (5), and description of interventions/comparisons/description of intervening factors (6) could not be logically applied to cross-sectional or observational studies. All studies awarded positive quality ratings needed to adequately address selection bias, make appropriate study group comparisons, clearly describe any interventions, and use valid and reliable measurements. To receive a “Yes” for criterion (7), “Were outcomes clearly defined and the measurements valid and reliable?”, the questionnaire needed to undergo a least three different types of expected psychometric validation, outlined by Parmenter and Wardle [27], as above. Meta-analysis was not possible due to heterogeneity in measures used to assess nutrition knowledge. 

## 3. Results

### 3.1. Study Selection

The original search yielded 331 results. After removal of duplicate and irrelevant records, 42 studies were retained for full-text review. An additional 11 records were identified through hand searching reference lists. Thus, a total of 53 full-text articles were screened for inclusion in the final review. Thirty-six of these met the inclusion criteria. The reasons for excluding the other articles included: the age of the participants being less than 17 years old (*n* = 5), inability to extract a mean score (*n* = 9), lack of adequate questionnaire description (*n* = 2), or failure to assess nutrition knowledge (*n* = 1) (Figure 1). A secondary search using the term ‘coach’ did not yield any additional relevant articles. 

### 3.2. Study Characteristics

The majority of the studies (*n* = 34) employed a cross-sectional design, with the remaining two [32,33] using a questionnaire to assess the effectiveness of an education program at two time points. Of the 36 included studies, 15 assessed nutrition knowledge in American college athletes [13,23,24,25,32,33,34,35,36,37,38,39,40,41,42]; two of these also collected data on coaches and athletic trainers, stratifying the results [23,24]. There were an additional four studies [38,43,44,45] that assessed the knowledge of coaches alone. Six studies assessed college athletes outside of the USA (three in Iran [15,20,46]; one each in India [17], Malaysia [21], and Nigeria [18]). Five studies [12,14,16,22,47] were conducted with elite athletes and three studies [48,49,50] assessed knowledge in recreational athletes. Five studies [15,24,33,34,45] did not report what sport the athletes played. Across the remaining studies, the other sports that were represented included: Australian football (AFL) [16], basketball [13,20,23,35,37,38,39,40,42,44], baseball [23,25,37,38,42], cross-country [13,35,41,42,44], cycling [50], football [13,20,23,35,37,38,44], golf [13,23,35,37,40], gymnastics [13,35,40,49], hockey [35,40,47], lacrosse [23,35,39], soccer [13,23,32,38,42], softball [13,19,35,37,40,42,44], running and/or track and field [14,23,25,35,37,42,44,48], rugby [12,22,47,51], swimming [13,18,32,35,37,52], tennis [35,37,38,42,43], and triathlon [50]. Participant numbers ranged from five [17] to 595 [46]. Most studies were mixed-gender (*n* = 19) [13,14,15,18,20,21,22,23,24,35,36,37,39,44,46,47,50,53]. There were a total of 5231 participants: 2307 males, 2170 females, and 754 where gender was not reported. The mean age ranges of coaches and athletes were 33.0 to 43.2 years and 19.0 to 35.2 years, respectively. No studies reported the nutrition knowledge of older athletes (master’s level) (Table 2).

### 3.3. Nutrition Knowledge Results

#### 3.3.1. Demographic Factors Related to Nutrition Knowledge Scores

Seven out of 11 studies that reported on prior nutrition knowledge found that higher levels of (general) education, previously undertaking a nutrition course, or currently majoring in nutrition studies correlated with higher nutrition knowledge scores [14,20,40,41,46,48,50]. Fifteen studies reported on male versus female scores, and 10 of these studies reported no significant difference [14,15,21,35,36,39,42,44,49,53]. All studies that assessed for differences between athletes from varying sports reported no significant differences in nutrition knowledge scores based on sport played [21,25,39,40]. Where reported, there was no significant difference in nutrition knowledge scores across National College Athletic Association (NCAA) divisions I, II, and III (ranked according to level of support and participation) [41,52].

#### 3.3.2. Nutrition Knowledge Scores of Athletes versus Comparison Group (within Studies)

Five studies included a non-athlete comparison group [21,22,34,48,50]. A study of American college athletes found that athletes had lower nutrition knowledge scores than a non-athlete comparison group consisting of 28% nutrition majors [34]; however, a study of college athletes in Malaysia found athletes had similar levels of knowledge when compared to non-athlete controls whose prior exposure to nutrition education was not reported [21]. Recreational athletes scored better than matched fitness class participants [48] and a matched community sample [50]. In contrast, a sample of elite athletes scored lower than both a matched community sample and a cohort of dietitians [22] (Table 2). 

Four studies also included athletes of various levels and/or both athletes and coaches [15,23,24,47]. University athletes in Iran were found to score better than non-university athletes [15] and elite athletes in New Zealand achieved higher scores than non-elite athletes [47]. Coaches scored better than athletes in the two studies that included both groups [23,24]. However, in all of these studies except the one comparing Iranian athletes [15], it was unclear whether the participants in various groups were comparable in terms of factors such as age, gender, and education (Table 2). 

#### 3.3.3. Comparison across Questionnaires (between Studies)

While a comparison of nutrition knowledge scores cannot be made across all studies due to the heterogeneity in measures and participants, some of the studies did use either the same tools or modified versions of such tools. The tool that Werblow et al. [40] developed for use in American college athletes was later modified and used in two other studies assessing similar groups [33,34]. Results in these studies were reasonably consistent at 68%, 68%, and 67%, respectively. The sports questionnaire developed by Zinn et al. [54], was used in three of the studies that assessed knowledge of coaches [44,45,51]. Scores observed in these studies were very similar, at 55%, 56%, and 56% respectively. The questionnaire developed by Zawila et al. [41] (for use in college runners) was based on a composite of two previous measures [40,48]. It was utilized by two other researchers assessing knowledge in American college athletes [36,52]. Three of the studies in non-USA college athletes [15,17,18] also used the tool by Zawila et al. [41] or one of the two original tools it was based upon. The results reported in the studies using these tools in American college athletes were 57%, 58%, and 72% and in Non-American college athletes were 54%, 39%, and 64%, respectively. Three of the five studies in elite athletes used a version of the “general nutrition knowledge questionnaire” [12,16,22]; scores in these studies were moderately disparate at 60.5%, 72.8%, and 65.3% respectively (Table 2).

### 3.4. Responses to Specific Nutrition Sub-Sections and Nutrition Questions

Given that there is a large degree of discrepancy in the question type and format across measures, scores reported as percentages provide little information regarding the actual knowledge (and gaps in knowledge) of participants. Therefore, in addition to reporting on the scores (% total correct) obtained in specific nutrition sub-sections (Table 2), we have provided a summary of nutrition sub-sections that were tested in each questionnaire (Table 3) and included a summary of responses to individual questions (Section 3.4.1, Section 3.4.2, Section 3.4.3, Section 3.4.4, Section 3.4.5, Section 3.4.6, Section 3.4.7, Section 3.4.8, Section 3.4.9 and Section 3.4.10).

#### 3.4.1. General versus Sports Nutrition Knowledge

The majority of studies assessed both general and sports nutrition knowledge. Four studies directly compared these nutrition sub-sections; scores were better in the sports nutrition knowledge compared to general nutrition knowledge section in two studies [16,33]; however, the opposite was true for the other two [47,48] (Table 2).

#### 3.4.2. Weight Management and Energy Balance

Eight studies reported on weight management and energy balance nutrition sub-section scores [14,17,20,23,38,39,45,51] (Table 2). Several authors also described responses to specific questions related to this topic. In many of the studies, athletes had a sound understanding of safe weight loss practices based on current recommendations. For example, in a study of recreational athletes, 92% of participants [48] disagreed that fasting is a good way to decrease fat and increase muscle. Similarly, 100% of swimmers in the study by Hoogenboom et al. [52] felt that skipping meals was not an acceptable way to lose weight. About 75% of college athletes in the study by Rosenbloom et al. [37] knew that eating carbohydrate would not “make them fat”. Nevertheless, misconceptions were evident; for example, 84% of female college athletes in the study by Collison et al. [34] and 92% of male and female college athletes in the study by Weeden et al. [39] agreed that “acidic foods such a grapefruit could assist with weight loss”. Likewise, Harrison et al. [47] found that 84% of elite and 63% of non-elite athletes disagreed with the statement “you can lose weight by decreasing your food intake”.

#### 3.4.3. Macronutrients

All studies but one assessed knowledge of carbohydrates [46]. Protein was not assessed in five studies [18,35,46,48,50]. Fat was not assessed in seven studies [18,33,35,40,42,46,48] (Table 3). Across these studies, there was no discernible pattern regarding related nutrition sub-section scores (% total correct) being above or below the overall mean nutrition score (% total correct) (Table 2). Several studies also reported on responses to individual items related to the energy density, role, sources, and requirements of macronutrients. 

*Energy density*: Devlin and Belski [16] found that only 22% of elite Australian Rules Football players were aware that fat is the most energy-dense macronutrient. Likewise, only 22% of U.S. college swimmers surveyed by Hoogenboom et al. [52], 28% of American college athletes surveyed by Collison et al. [34], and 18% of American college coaches in the study by Corley et al. [53] knew that carbohydrates and protein have the same amount of energy per gram. 

*Role*: Sixty-nine per cent of Nigerian athletes in the study by Folasire et al. [18], 98% of elite athletes surveyed by Hamilton et al. [14], and 64% of college athletes in the study by Rosenbloom et al. [37] agreed with a statement indicating that that foods rich in carbohydrate should be the main source of energy. Rosenbloom et al. [37] and Rash et al. [36] also reported that about 46% and 40% of College American college athletes thought that protein was a source of fuel for muscles or believed protein was a good source of “immediate” energy. However, none of the coaches surveyed by Corley et al. [53] subscribed to similar beliefs. 

*Sources*: Only 42% of elite athletes surveyed by Hamilton et al. [14] correctly answered questions on sources of saturated and unsaturated fat. Similarly, less than one-quarter of elite Australian Rules Football players in the study by Devlin and Belski [16] selected dairy as a source of saturated fat and less than one-fifth were aware of the saturated fat content of margarine and red meat. On the other hand, many of the elite Australian Rules Football players were able to identify foods that were low in both protein and carbohydrates [16], and 100% of coaches surveyed by Corley et al. [53] knew that sources of dietary carbohydrates include breads, crackers, and pastas. 

*Requirements*: In the studies by Shifflett et al. [24] and Hoogenboom et al. [52] only 21% and 25% of college athletes, respectively, knew what proportion of energy should come from fat; slightly more, 41%, knew the proportion of energy that should come from protein [24,52]. Similarly, in the study by Weeden et al. [39], when asked what carbohydrate range was endorsed by experts, 53% of college athletes selected a value below the current recommendations. 

*Quality*: Only 50% of elite and 26% of non-elite athletes surveyed by Harrison et al. [47] disagreed that “athletes who are vegetarians perform as well as non-vegetarian athletes”. This belief was also reported by Rash et al. [36], where 82% of American college athletes believed that vegetarian athletes needed protein supplementation. Devlin and Belski [16] and Hamilton et al. [14] found that 80% of elite Australian Rules Football players [16] and 55% of elite athletes in New Zealand, respectively were aware that most of the fats in our diet should be unsaturated. In relation, all college coaches surveyed by Corley et al. [53] agreed that plant oils are healthier than animal fats.

#### 3.4.4. Micronutrients

All studies except five [33,35,37,40,42] assessed knowledge of micronutrients (Table 3) and eight studies reported on micronutrient sub-section scores [14,15,17,23,36,38,47,51] (Table 2). Arazi and Hosseini [15] reported that the mean scores for the sections covering knowledge of “Vitamins” and “Calcium and Iron” were 61% and 56%, respectively. These scores were higher than the overall mean of 54%. These results were echoed in the study by Zawila et al. [41], where questions on iron were answered correctly by more than 70% of runners, and in the study by Hamilton et al. [14], where most swimmers answered questions on vitamin C (85%–100%) and iron deficiency (98%) correctly. In contrast, Rash et al. [36] reported that college athletes’ scores on both vitamin C and vitamin E questions were below the overall mean scores. Information on responses to specific questions on the role, sources, and requirements of micronutrients was also included in some studies. 

*Role*: Only 17% of college athletes surveyed by Weeden et al. [39] could identify the differences between fat and water soluble vitamins. Sixty-seven percent of college males [37] and 72% of college females [34] surveyed knew that vitamins do not provide extra energy, but 56% of a different sample of college athletes [36] and 56% of a sample of Nigerian college athletes [18] thought this statement was true. Just 19% of elite and 9% of non-elite athletes surveyed by Harrison et al. [47] selected “false” to the statement, “vitamin B-complex helps you to recover faster”.

*Sources*: Ninety-six percent of recreational runners in the study by Barr [48] knew that bananas and avocados are good sources of potassium, 89% knew that bread is not a good source of calcium, and 56% knew “apples are a good source of vitamin C”. On the contrary, many male college athletes in the study by Shoaf et al. [25] thought that milk was high in iron, and many triathletes in the study by Worme et al. [50] believed that iron was the main nutrient found in spinach.

*Requirements*: In the study by Zawila et al. [41], 60% of female athletes thought that calcium needs could be met by having just two glasses of milk. On the other hand, all female recreational marathon runners surveyed by Barr [48] knew that women need more iron than men and 69% of coaches surveyed by Corley et al. [53] correctly selected “false” for the statement “female athletes need more B vitamins than any other athlete”. 

#### 3.4.5. Supplementation

Twenty-one studies included questions on supplementation [14,16,17,20,21,23,24,25,34,36,37,38,39,41,43,44,45,47,51,52,53] (Table 3). Five studies reported on supplementation sub-section scores [14,20,23,38,45]; correct responses to this section were high in some studies, but low in others (Table 2). Many authors also provided information on how individual items pertaining to ergogenic aids, vitamins, and minerals, as well as protein supplementation, were answered.

*Ergogenic aids*: Shoaf et al. [25] found that supplement questions were answered correctly by 87% of male college athletes; items on creatine were answered correctly more than 70% of the time. Eighty-two percent of elite athletes surveyed by Hamilton et al. [14] knew that steroids are not safe, but only 26% were aware that caffeine can help extend performance.

*Vitamins and Minerals*: In the paper by Hamilton et al. [14] only 29% of elite athletes thought vitamin C supplements help fight colds. However, in other studies misconceptions were common: 72% of college coaches surveyed by Corley et al. [53] thought all vegetarian athletes required zinc supplementation; 50% of elite athletes surveyed by Hamilton et al. [14] believed multivitamins would increase energy levels and 45% though they were ‘vital for topping up performance’. In relation, 76% of college athletes surveyed by Rash et al. [36] felt a general vitamin and mineral supplement was needed daily, 53% believed that they needed vitamin C supplements to boost immune function, and 56% believed that vitamin E supplementation was needed to protect red blood cells (RBC) from oxidative damage and to promote oxygen transport to RBC. In contrast, 89% of coaches in the study by Corley et al. [53] disagreed with the statement that vitamin pills could be taken in unlimited amounts and 72% knew that vitamin pills are not needed if a well-balanced diet is consumed.

*Protein*: Jessri et al. [20] reported that 43% of female and 47% male Iranian college athletes believed all athletes needed protein supplementation; and, while only 34% of elite athletes in the study by Hamilton [14] disagreed that “protein supplements build larger muscles and make you stronger”, 79% of college coaches surveyed by Corley et al. [53] selected “false” for a similar statement. One-third of coaches surveyed by Zinn et al. [51] felt that protein powder was essential for weight loss. 

#### 3.4.6. Fluids

Twenty-three studies asked questions about fluids [14,16,17,18,19,20,21,23,24,36,37,39,41,42,43,44,45,47,48,51,53] (Table 3). Six studies reported on fluid (or hydration) sub-section scores [17,20,23,38,39,51]. In some studies, the scores (% total correct) in this section were above the overall mean; however, in others they were below the overall mean (Table 2). Several studies also reported on the frequency with which individual items related to the need for fluid, fluid timing, and fluid type were answered correctly.

*Need for fluid*: Weeden et al. [39] found that 92% of USA college respondents were able to identify the importance of water in body temperature regulation, and 97% knew the best sources of electrolytes. Rosenbloom et al. [37] found that 93% of college athletes agreed that dehydration decreases performance. Similarly, Corley et al. [53] found that 89% of coaches were aware that fluids are required to prepare for sweat losses.

*Timing of fluid ingestion*: Harrison et al. [47] reported that 79% of elite and 68% of non-elite athletes knew that you should drink during exercise lasting over one hour, and that 65% of elite 45% of non-elite athletes knew you should drink before exercise. Likewise, about 95% of college athletes in the study by Rosenbloom et al. [37] and 94% of college coaches in the study by Corley et al. [53] agreed ingestion of water was important before, during, and after exercise.

*Type of fluid*: Jessri et al. [20] stated that only 1.3% of female and 0.6% of male Iranian college athletes could identify the amount of carbohydrate a sports drink should contain. While Folasire et al. [18] reported that 59% of Nigerian university athletes knew that sports drinks were best to replace fluids, only 22% of American college athletes in the study by Rosenbloom et al. [37] agreed that they are better than water. 

#### 3.4.7. Pre-Competition Meal

Seventeen studies included questions on the pre-competition meal [14,16,18,19,20,21,23,25,35,37,43,44,45,47,48,51,53] (Table 3). Only one author [45] reported on a pre-competition sub-score, indicating that scores in this section were above the overall mean scores (Table 2). Two studies [34,37] also reported on responses to individual items relevant to pre-competition nutrition. In the study by Collison et al. [34], 72% of athletes selected “false” for “carbohydrate loading will enhance performance in all events of 1 h or less”, 95% agreed that “high carbohydrate meals require 2 to 3 h to be emptied from the stomach”, and 66% agreed that “high-fat meals should not be eaten 2–3 h before competition”. Conversely, Rosenbloom et al. [37] found that 63% of male and 71% of female college athletes thought sugar eaten before an event will adversely affect performance. 

#### 3.4.8. Nutrition during Competition

Only one paper included questions on nutrition during competition [48] (Table 3). There was no specific information on how these questions were answered. 

#### 3.4.9. Recovery Meal

Seven studies included questions on the recovery meal [16,20,24,44,45,48,51] (Table 3). No studies provided a summary of specific questions on recovery. However, both Danaher and Curley [45] and Zinn et al. [51] reported on recovery as a nutrition sub-section. Coaches performed poorly in the former study, but well in the latter (Table 2).

#### 3.4.10. Alcohol

Only one paper assessed knowledge of alcohol [16] (Table 3). While 89% of elite Australian Rules Football players in this study were able to identify safe alcohol consumption guidelines, only 33% selected the correct alcoholic beverage when asked which was an example of a “standard drink”, and just 38% correctly answered a question regarding grams of ethanol in a “standard drink” [16].

### 3.5. Quality Assessment of Included Articles/Risk of Bias

Quality analysis was performed for all studies that met inclusion criteria, and results of quality analysis did not alter decisions about inclusion. Only one of the studies received a positive (“Yes”) rating [49]. All of the other included studies received a neutral rating indicating moderate study quality. Ratings were mostly affected by the lack of inclusion of a comparison group, and use of tools that had not undergone adequate validation. In many cases, participant characteristics were not well described.

### 3.6. Quality Assessment of Tools Used

The comprehensiveness scores ranged from one to 10 (Table 3). None of the studies used a questionnaire that covered all 11 nutrition sub-sections that were deemed relevant. Three studies received scores of less than or equal to two; however two of these [35,42] only aimed to test a single nutrition sub-section of nutrition knowledge—carbohydrates and hydration respectively. The third [15] received the very low score because it was unclear what was and was not tested. Thirteen studies [14,16,20,21,23,24,39,41,44,45,47,51,52] covered eight or more (that is, more than 75%) of the relevant nutrition sub-sections.

The validation scores ranged from zero to six out of six (Table 4). Four studies made no mention of validation [21,35,37,44], scoring zero. Two authors [16,41] used a combination of two previously validated questionnaires but did not perform any assessment of the composite tool, also scoring zero. Eight studies used questionnaires that underwent just one type of psychometric analysis [23,36,41,42,47,48,50,53]. Two authors [36,52] used the questionnaire by Zawila et al. [41] with minor modification, assessing the tool for face and content validity, scoring two. Three studies [13,15,49] used questionnaires that had undergone five out of the six possible validation procedures. Just one study [22] scored 6, the maximum amount of available points. All of the studies that scored five to six for the validation score utilized the “general nutrition knowledge questionnaire” [55].

## 4. Discussion

### 4.1. Study Selection and Study Characteristics

The aim of this review was to summarize current levels of knowledge in athletes (aged 17 years and older) and coaches, and to provide a detailed assessment of the quality of the tools used to assess nutrition knowledge. Our search yielded 36 studies that met the inclusion criteria; 10 [15,16,17,18,19,21,22,32,39,56] of the studies on athletes were published after 2010, when a previous complementary review on athletes was conducted [7]; there were also an additional seven [35,42,43,44,45,51,53] relevant studies that had been not included in the aforementioned review due to differing inclusion criteria. Males and females tended to be equally represented. The majority of research has been conducted with American college athletes [13,19,23,24,32,33,34,35,36,37,40,41,42,52], presumably because they are easy to recruit. Our search did not retrieve a single paper on the knowledge of elite athletes in North America; this is surprising considering the scope of elite athlete leagues in this region. Likewise, while a broad range of sports are covered across the literature, there was only one study in netball players [47], and athletes from many other popular Commonwealth sports, such as cricket, were underrepresented. There is a need for research that is representative of various types of athletes. A better understanding of specific athletes who may have poor knowledge will allow professionals working with these populations to advocate for increased education and support.

### 4.2. Quality Assessment of Included Articles and Quality Assessment of Tools Used

Akin to previous complimentary reviews [7,56], a key finding of this review was that there were issues with quality of the included studies, and the questionnaires used to assess nutrition knowledge were inadequately validated. Despite recommendations made in a 2011 review by Heaney et al. [7] that studies assessing nutrition knowledge should collect and report demographic data, include comparison groups, and use validated tools, the quality ratings of newer studies (i.e., those published since 2010) do not appear to be higher than the ratings of older studies. In relation, even though the validity of tools used to assess nutrition knowledge have been questioned in previous reviews [7,56], no new tool has been developed and validated. This is likely because the time and resources required for tool development can be prohibitive [27]. Most studies that did receive a high validation score (for the measurement instrument) used the “general nutrition knowledge questionnaire” [55], and since this does not assess sports nutrition knowledge, these studies received low scores on the comprehensiveness rating. There were a number of issues related to the content included in the tools. Only one of the questionnaires asked questions on alcohol, which is an important topic given the drinking culture among sports people [57,58]. There are also several important considerations in regards to the relevance/accuracy of some of the items. All of the questions on carbohydrate recommendations state requirements as percent total macronutrient contribution, however more recent consensus statements provide recommendations in grams per kilogram of body weight per day [2]. Furthermore, experts may no longer agree with the “correct” answers to some questions, for instance, Collison et al. [34] indicated that tea, cola, and coffee were NOT the best pre-athletic event beverages but it could be argued that these may be beneficial due to their caffeine content, which is a known ergogenic aid for some athletes [59]. Several other examples of outdated questions exist. Many of these are in relation to hydration, specifically with regards to thirst as an indicator for fluid needs [60]. It is axiomatic that the strong consensus regarding dietary strategies for optimal athletic performance should be reflected in questionnaires designed to assess nutrition knowledge [7]. Researchers developing tools to assess the nutritional knowledge of sports people should ensure they address the aforementioned limitations. 

Another important factor to consider is whether tools are validated for the population they are being used with [7,61]. The questionnaire developed by Zinn, et al. [54] was used in two other studies in coaches [20,44], with just a 1.5% difference in scores between them [54,55], indicating it may have good validity in this cohort. On the other hand, the range of scores on the “general nutrition knowledge questionnaire” [12,13,16,22,49] was quite large (51.5%–74.2%), even when comparing across similar athletic levels (e.g., scores across studies in elite athletes using this tool ranged from 60.5% to 72.8%). This tool was developed for a British audience and has been modified for several other population groups including the Australian population. Interestingly, the highest scores on the “general nutrition knowledge” tool were achieved in British cohorts of elite and recreational athletes [12,49], middle scores in an Australian sample of elite athletes [22] and the lowest scores in a sample of college athletes in the USA [13]. While it is certainly possible that this variation was due to factors such as athletic level or age [7,22] it is worth considering that this tool was not culturally appropriate for North American athletes.

### 4.3. Nutrition Knowledge Results

Given that all studies received a neutral quality rating and that many of the measurement tools used were inadequately validated, it is difficult to definitively comment on the both the current status of nutrition knowledge of athletes and coaches, and the factors that may influence nutrition knowledge. One consistent finding was that education impacts nutrition knowledge; it is therefore important that questionnaires cater to various literacy levels (e.g., by including pictures) so that scores are reflective of actual nutrition knowledge, rather than literacy in general. It appears nutritional knowledge may also be affected by athletic level, and that coaches’ knowledge is better than athletes’. Theoretically, it is plausible that elite athletes have greater access to resources and therefore higher levels of knowledge; likewise, it is likely that American college athletes have more support and funding than non-USA college athletes. In contrast to previous findings [7], our review did not suggest that gender or the type of sport played affects nutrition knowledge. Likewise, findings comparing athletes to non-athlete comprising groups were inconsistent. More quality research is needed to ascertain whether these associations are confounded by demographic factors, study quality, and questionnaire quality.

### 4.4. Responses to Specific Nutrition Sub-Sections

Scores reported as a percentage are fairly arbitrary unless they are being used to compare different groups within the same study, or changes to the same group over time. While several authors have suggested various “cutoff” points that signify adequate levels of knowledge (e.g., Torres-McGehee et al. [23] stated that >75% was indicative of adequate knowledge), these values add little meaning. It cannot be assumed that a questionnaire with 11 items covering a few relevant nutrition sub-sections is equivalent to a 76-item questionnaire that addresses multiple topics of general and sports nutrition knowledge. Therefore, we synthesized responses to nutrition sub-sections and individual questions. It is clear that there is considerable discrepancy between studies. In many cases, participants scored poorly in a section in one study, and well in the same section in another study. The lack of consistency makes drawing conclusions about gaps in knowledge difficult. Overall, however, it appears that understanding of the following topics was poor: energy density, the need for vitamin and mineral supplementation amongst athletes, the role of protein in muscle synthesis, sources of fat, and the need for protein supplementation among athletes trying to lose weight and athletes who follow a vegetarian diet. Awareness of areas of knowledge that require improvement is an important consideration when designing interventions (one-on-one) and education programs aimed at improving nutrition knowledge of athletes. Specific gaps in knowledge cannot be ascertained from nutrition knowledge results that are reported as percentage total correct responses. Researchers should consider the ways in which knowledge results are reported. It should be clear what topics (nutrition sub-sections) of knowledge were tested and whether the tool used was able to identify particular nutrition concepts that were not well understood. 

## 5. Limitations

A major limitation of this review is that meta-analysis of scores across studies was not possible. This was due to the relatively small amount of included studies, the under-representation of various sporting disciplines and levels, the lack of representativeness within studies, the heterogeneity of participants across studies, and the heterogeneity of the measures used to assess nutrition knowledge. 

There are also limitations related to how the tools have been rated (Table 4). It was often unclear how information collected during “pre-testing” was actually used to modify the questionnaire being piloted. Firstly, judgements were hindered by the vague description provided of the type and extent of validation that has been performed. For example, Sedek and Yih [21] stated that they used a questionnaire that has been validated by Paugh [62]. In fact, this was an unpublished thesis that only assessed Cronbach’s alpha (α = 0.56). Secondly, validation scores were based on the steps outlined by Parmenter and Wardle [27], but their protocol does not describe factor analysis or Rasch analysis. Factor analysis assesses a scale’s dimensionality, and therefore can be used to decide whether Cronbach’s alpha is appropriate [30,63]. Rasch analysis is an Item Response Theory (IRT) technique, which allows for shorter scales, with multiple response formats to be developed [64]. Finally, although a point was awarded if a topic was deemed to be covered in adequate detail (Table 4), there was still a large variety in the depth and detail in which nutrition sub-sections were covered. For instance, while the questionnaires used by Collison et al. [34] and Zinn et al. [54] both covered supplements, the former only included two questions on the use of diuretics and multivitamins and the latter tested knowledge of creatine, hydroxy-beta-methlybutyrate (HBM), micronutrient supplementation, and appetite suppressants. The quality of individual items was not taken into account when designing the comprehensiveness score. The issues with individual items are beyond the scope of this review. They include, but are not limited to, ambiguous wording and reference to outdated recommendations as described in Section 4.2.

## 6. Conclusions

The quality and heterogeneous nature of the included studies and of current measures used to assess nutrition knowledge make assessment of general and sports nutrition knowledge in athletes and coaches difficult to ascertain. Specific gaps in knowledge also remain largely unclear, although analysis of individual items indicates that it is likely that energy density, supplementation, and the role of protein are commonly misunderstood topics. It is possible that there is a relationship between gender, athletic level (e.g., elite) and nutrition knowledge; however, more high-quality research is needed to confirm these assertions.

Nutrition knowledge is a modifiable determinant of dietary behavior, and therefore has the potential to have a significant impact on athletic performance. Accordingly, there is a need for additional high-quality research on this topic. However, the low quality of current measures of nutrition knowledge means that none of the currently available tools can confidently be endorsed for use in future studies. It is therefore the recommendation of the authors that a new, universal, up-to-date, validated measure of general and sports nutrition knowledge be developed. Such a tool should consider health literacy, cultural appropriation, and current consensus recommendations regarding nutrition for optimal athletic performance, and should undergo rigorous validation that includes techniques from within an item response theory framework. Moreover, the questionnaire should have the capacity to report a knowledge “profile”, outlining gaps in knowledge and areas where knowledge is well understood. A quality tool would allow more robust assessment of knowledge of both athletes and coaches, having utility in clinical practice, the development and evaluation of education programs, and research in the field. Over time this new tool would allow more robust comparisons across various groups to be made.

## Figures and Tables

**Table 1:** Eligibility criteria.

Included	Excluded
Original research (cross-sectional, observational, randomized controlled trials)	Abstracts, conference posters, reviews, and unpublished theses
2. Athletes (aged 17 years and older) and coaches of adult athletes (recreational, elite)	2. Adolescent athletes, all non-athletes other than coaches
3. English language studies	3. Non-English language studies
4. Studies reporting a quantitative measures of nutrition knowledge that could be converted into a single ‘score’ (% total correct)	4. Studies on nutrition attitudes, behavior, habits, or intake; studies where a mean nutrition knowledge score could not be determined
5. Studies that described the tool used to assess knowledge including number of items, content and question response-categories	5. Studies where it was unclear what (and how) the tool used actually measured nutrition knowledge

**Table 2:** Nutrition knowledge of athletes and coaches.

References	Participant Characteristics				Questionnaire					
Author, Year, Country	Athletic Level	Sport Played	*N* (Gender)	Mean Age (Years) ± SD	Questionnaire Used/Item Generation and Number of Questions	Type of Questions	Mean Correct Nutrition Knowledge Score (%) ± SD	Nutrition Sub-Sections with Scores above Average Compared to the Total Mean Score within the Same Study (% ± SD Where Available)	Nutrition Sub-Sections with Scores below Average Compared to the Total Mean Score within the Same Study (% ± SD Where Available)	Quality Rating
Abood et al. [32], 2004, USA	College	Soccer, Swimming	*n* = 30 (F)	19.5 (SD NR)	Self-developed; *n* = 42	True/False	**68.5**	NR	NR	Neutral
Alaunyte et al. [12], 2015, UK	Elite	Rugby	*n* =21 (M)	25 ± 5	Existing Questionnaire(A–C of GNKQ); *n* = 28	Multi-Choice, Open-Ended, Less/More/Not Sure/Same	72.82 ± 6.11	Recommendations made by experts (85.7 ± 13.0)	Food groups (71.2 ± 7.2) and Making healthier Food Choices (69.5 ± 14.0)	Neutral
Arazi and Hosseini [15], 2012, Iran	College/‘Non-College’	NR	*n* = 250 (130 M; 120 F); 121 College, 129 Non-College	College M: 24.71 ± 2.3, College F: 23.61 ± 2.10, Non-College M: 23.42 ± 1.8, Non-College F: 21.49 ± 2.8	Modified Questionnaire (Zawila et al. 2003); *n* = 40	True/False	**54.0**	Vitamins (61.2), Calcium and Iron (56.48), Weight Loss (57.95)	Macronutrients (50.7), Fiber (52.3), Sports Nutrition (49.74), General Nutrition (49.97)	Neutral
Azizi et al. [46], 2010, Iran	College	Sport Olympiad (range of sports)	*n* = 595 (298 M; 297 F)	M: 22.8 ± 1.9, F: 21.8 ± 1.8	Self-developed; *n* = 15	Strongly agree/agree/neutral/disagree/strongly disagree	* **58.9** (M: 52.36 ± 6.2; F: 54.3 ± 6.3)	NR	NR	Neutral
Barbaros-Tudor et al. [43], 2011, Croatia	Coaches	Tennis	*n* = 58 (50 M; 8 F)	33 ± 10. 8	Self-developed; *n* = 40	True/False	68.9 (SD not reported)	NR	NR	Neutral
Barr [48], 1987, USA	Recreational	Marathon runners	*n* = 104 (F) And *n* = 105 fitness class participants (F)	NR; 1.0% <20, 40.8% 20–29, 43.7% 30–39, 14.6% >40 years	Self-developed; *n* = 87	True/False/Don’t Know	Athletes: 50.1 *; Fitness class participant: 42.6 * (SD not reported)	Knowledge about general nutrition (50.9)	Knowledge about sports nutrition (48.3)	Neutral
Botsis and Holden [44], 2015, USA	Coaches	Volleyball, Softball, Cross-Country, Track and Field, Football, and Basketball	*n* = 21 (16 M; 5 F)	NR	Existing Questionnaire (Zinn et al. 2006); *n* = 88	Agree/Disagree/Unsure, Multi-Choice	55 (SD not reported)	NR	NR	Neutral
Collison et al. [34], 1996, USA	College	NR	*n* = 51 (F) And *n* = 49 (F) comparison group	19.4 ± 1.2	Modified Questionnaire (Werblow et al., 1978); *n* = 35	Agree/Disagree	Athletes: 68.3 Comparison group: **77.1**	NR	NR	Neutral
Condon et al. [35], 2007, USA	College	Ice hockey, Lacrosse, Basketball, Track and Field, Softball and Tennis	*n* = 165 (63 M; 102 F)	20 ± 1.3; M: 20.3 ± 1.5, F: 19.7 ± 1.1	Self-developed; *n* = 8	True/False, Open-ended, Multi-choice	**50.0**	NA	NA	Neutral
Corley et al. [53], 52, USA	College Coaches	Track and Field, Cross-Country, Swimming, Tennis, Basketball, Gymnastics, and Golf, and Men’s Football, and Men’s Wrestling	*n* = 105 (75 M; 30 F)	35	Self-developed; *n* = 15	True/False/Not Sure	* **60.09**	NR	NR	Neutral
Danaher and Curley [45], 2014, Canada	College Coaches	NR	*n* = 5 (NR)	NR	Modified existing Questionnaire (Zinn et al., 2005); *n* = 95	Agree/Disagree/Unsure, Multi-Choice	**56.3**	Training diet (58.1), Pre-competition diet (58.1), weight loss and weight gain (57.5)	Fluid (49.5), Recovery diet (50.6), Dietary Supplements (53.8) * not statistically analyzed	Neutral
Davar [17], 2012, India	College	Hockey	*n* = 30 (F)	19.9 ± 2.7	Modified existing Questionnaire (Zawila et al. 2003); *n* = 61	True/False, Open-Ended	38.8 (SD not reported)	Protein (41.1), Fats (53.5), Vitamins (39.3), Minerals (43.8), Hydration (51.6)	Energy (25.9), Carbohydrates (37.7), Weight management (32.3), Sports nutrition (36.5), Fiber (21.4)	Neutral
Devlin and Belski [16], 2015, Australia	Elite	Australian Football (AFL)	*n* = 46 (M)	23.5 ± 2.8	Modified Existing questionnaire (GNKQ + Shifflet); *n* = 123	Multi-Choice, Open-Ended, Less/More/Not Sure/Same	60.5 (SD for % score not reported)	Sources of nutrients (60.9), Sports Nutrition Knowledge (61.7)	Dietary recommendations (60.0), Sources of nutrients (57.0)	Neutral
Dunn et al. [13], 2007, USA	College	Basketball, Golf, Gymnastics, Softball, Swimming, Soccer, Tennis, Cross-country, Volleyball, Football	*n* = 190 (92 M; 98 F)	19.0	Existing Questionnaire (GNKQ); *n* = 124	Multi-Choice, Open-Ended, Less/More/Not Sure/Same	51.5 ± 13.57	Dietary recommendations (59.3), Food groups (54.4)	Dietary recommendations (60.0), Sources of nutrients (57.0)	Neutral
Folasire et al. [18], 2015, Nigeria	College	Ball-games’, Racquet, ‘Combat sports’, Swimming	*n* = 110 (63 M; 47 F)	22.06 ± 2.39	Self-developed (used items from Zawila et al. 2003 and Supriya et al. 2013); *n* = 14	Yes/No/Not Sure	64.3 (SD not reported)	NR	NR	Neutral
Grete R et al. [19], USA	College	Softball	*n* = 185 (F)	NR	Self-developed; *n* = 80	Likert-Scale	* **57.1**	NR	NR	Neutral
Hamilton et al. [14], 1994, New Zealand	Elite	Distance Runners	*n* = 53 (41 M; 12 F)	24 ± 6	Self-developed; *n* = 48	Multi-choice	**64.0**; General M: 70 ± 14, General F: 78 ± 14, Sports M: 50 ± 16; Sports F: 58 ± 11	Vitamin C, energy and fiber (85.0–100), iron deficiency (98.0), recommended methods for weight loss (89.0), coronary heart disease (94.0–96.0). Protein as a fuel source, high carbohydrate foods and energy sources for vigorous exercise (74.0–98.0)	Foods high in saturated fat (42.0) and unsaturated fat (25.0), and changes in energy requirements with age (47.0). Carbohydrate loading, recommended ratio of dietary energy sources and ergogenic aids (6.0–17.0)	Neutral
Harrison et al. [47], 1991, New Zealand	Elite/‘non-elite’	Field Hockey, Basketball, Powerlifting, Netball (F only), Rugby Union (M only)	*n* = 122 (69 M; 53 F); 69 elite, 53 non-elite	23.75	Self-developed; *n* = 28	True/False/Not Sure, Open-Ended	Elite: 67.0 ± 12; non-elite: 56.0 ± 12	Energy sources (77.0 for elite)	Vitamins (9.0–50.0)	Neutral
Hoogenboom et al. [52], 2009, USA	College	Swimmers	*n* = 85 (F)	19 ± 1.6	Existing Questionnaire (Zawila et al., 2003); *n* = 76	Strongly agree/agree/neutral/disagree/strongly disagree	* 72.0 (SD not reported)	NR	NR	Neutral
Jessri et al. [20], 2010, Iran	College	Basketball, Football	*n* = 207 (109 M; 98 F)	21.8 ± 1.3	Modified existing Questionnaire (Zinn et al., 2006); *n* = 88	Agree/Disagree/Unsure, Multi-Choice	33.2 ±12.3	Nutrient type (36.75), Weight Control (33.35)	Fluid (33.05), Supplements (30.35)	Neutral
Kunkel et al. [33], 2001, USA	College	NR	*n* = 32 (F)	NR	Existing Questionnaire (Werblow et al., 1978); *n* = 31	Multi-choice, Agree/Disagree/Not Sure	66.7 ± 8.3	Sports Knowledge questions (66.3)	General Knowledge questions (54.5)	Neutral
Nichols et al. [42], 2005, USA	College	Soccer, Basketball, Tennis, Cross-country, Track, Baseball, Softball, and Volleyball	*n* = 139 (62 M; 77 F)	19.8 ± 1.5; M: 20.1 ± 1.6, F: 19.6 ± 1.4	Self-developed; *n* = 17	True/false	**81.8**	N/A	N/A	Neutral
Rash et al. [36], 2007, USA	College	Track and Field	*n* = 113 (61 M; 53 F)	M:19.3 ± 1.2, F: 19.1 ± 1.1	Existing Questionnaire (Zawila et al., 2003); *n* = 76	Strongly agree/agree/neutral/disagree/strongly disagree	58.3 ± 13	Carbohydrates (75.4), Vitamins and Minerals (62.7)	Protein (54.7), Vitamin E (45.0), Vitamin C (38.4)	Neutral
Raymond-Barker et al. [49], 2007, England	Recreational	Endurance athletes (runners, cyclists, triathlon), and gymnasts	*n* = 59 (F)	33.88 ± 9.74	Modified existing Questionnaire (GNKQ); *n* = 110	Multi-Choice, Open-Ended, Yes/No/Not Sure, Agree/Disagree/Not Sure	**74.2**	NR	NR	Positive
Rosenbloom et al. [37], 2002, USA	College	Football, Track and Field, Baseball, Swimming, Basketball, Tennis, Golf, Softball, Volleyball	*n* = 328 (237 M; 91 F)	M: 19.0 ± 2.7, F: 19.0 ± 1.3	Self-developed; *n* = 11	Agree/Disagree/Don’t Know	52.7 (SD not reported)	NR	NR	Neutral
Sedek and Yih [21], 2014, Malaysia	College	Futsal, Cricket, Pencak Silat, Volleyball, Silat Cekap, Taekwondo	*n* = 100 (50 M; 50 F), And *n* = 100 non-athletes (50 M; 50 F)	20.8 ± 1.8	Modified existing Questionnaire (Paugh et al., 2005); *n* = 29	Strongly agree/agree/neutral/disagree/strongly disagree	* Athletes: 83.7 ± 6.84 Non-athletes: 83.5 ± 6.23	NR	NR	Neutral
Shifflett et al. [24], 2002, USA	College Athletes and	NR	Athletes: *n* = 65 (12 M: 53 F); Coaches: *n* = 68 (39 M; 29 F)	Athletes: M: 20.0 ± 1.50; F: 20.0 ± 2.00; Coaches: M: 43.10 ± 9.7; F: 37.6 ± 9.4	Self-developed; *n* = 19	Multi-Choice, Open-Ended	**Athletes: 55.0; Coaches: 60.8**	NR	NR	Neutral
Shoaf et al. [25], 1986, USA	College	Baseball, Football, Track	*n* = 75 (M)		Self-developed; *n* = 25	Multi-Choice	43.8 (SD not reported as %)	NR	NR	
Smith-Rockwell et al. [38], 2001, USA	College Coaches	Baseball, Basketball, Cheerleading, Football, Cross-Country, Lacrosse, Rowing, Soccer, Swimming, Tennis, Track and field Volleyball, Wrestling	*n* = 53 (Gender NR)	34.2 ± 9.7	Self-Developed; *n* = 19	True/False/Multi-Choice	**67.0**	Weight control (71), Nutrition supplements (90), Other topics: fluids, amenorrhea, sources of nutrition information (92)	Macronutrients (51), Micronutrients (53)	Neutral
Spendlove et al. [22], 2012, Australia	Elite	Surf lifesaving, Rugby League	*n* = 175 (76 M; 99 F) And *n* = 116 community members And *n* = 53 dietitians	18.9 ± 4.9	Existing Questionnaire (GNKQ) and Modified existing questionnaire (R-GNKQ); R-GNKQ: *n* = 90	Multi-Choice, Open-Ended, Less/More/Not Sure/Same	Athletes: R-GNKQ—65.3 (95% CI 8.3, 8.8); Community: 65.4 (95% CI 8.2, 8.9) Dietitians: 77.7 (95% CI 9.5, 10.8)	GNKQ: Dietary recommendations (65.4), Sources of Nutrients/Food groups (60.9), Choosing Everyday foods (60.0); R-GNKQ: Dietary recommendations (73.4, Choosing everyday foods 68.0)	GNKQ: Diet-disease relationship (57.6) GNKQ: Sources of nutrients/food groups (62.1), Diet-disease relationship (48.9)	Neutral
Torres-McGehee et al. [23], 2012, USA	College Athletes and College Coaches’	Baseball, Basketball, Cheerleading, dance, Equestrian, Football, Golf, Ice-Hockey Lacrosse, Soccer, Swimming & Diving, Tennis, Track and Field, Volleyball, and Wrestling	Athletes: *n* = 185 (Gender NR); Coaches: *n* = 131 (Gender NR); Athletic trainers: *n* = 91; Strength and Conditioning coaches: *n* = 71	NR	Self-developed; *n* = 20	Multi-Choice	Athletes: 54.9 ± 13.5 Coaches: 65.9 ± 14.3 Athletic trainers: 77.8 ± 10.3 Strength and Conditioning Coaches: 81.6 ± 10.3	Supplements and performance (66.3 ± 19.9)	Micronutrients and Macronutrients (51.8 ± 20.3), Weight management and Eating disorders (47.0 ± * 21.9), Hydration (54.7 ± 24.2)	Neutral
Weeden et al. [39], 2014, USA	College	Male Basketball, Football, Tennis, Track and Field, and Female, Basketball, Golf, Soccer, Tennis, Track and Field and Volleyball	*n* = 174 (86 M; 88 F)	20.0 ± 1.4	Self-developed; *n* = 24	Yes/No/Not Sure	56.4 ± 13.4	Hydration (80.0)	Weight management (32.0), dietary supplements (36.0)	Neutral
Werblow et al. [40], 1978, USA	College	Softball, Track and Field, Gymnastics, Basketball, Field Hockey, Tennis, Swimming, Diving, Volleyball, Golf	*n* = 94 (F)	NR	Self-developed; *n* = 31	Strongly agree/agree/neutral/disagree/strongly disagree	* 67.74 (SD not reported)	NR	NR	Neutral
Worme et al. [50], 1990, USA	Recreational	Triathlon	*n* = 71 (50 M; 21 F) And *n* = 28 non-athletes (21 M; 17 F)	35.3	Self-developed; *n* = 20	True/False	Athletes: 54.2 ± 2.0, Non-athletes: 56.5 ± 2.3	NR	NR	Neutral
Zawila et al. [41], 2003, USA	College	Cross-country runners	*n* = 60 (F)	19.8 ± 1.04	Self-developed; *n* = 76	Strongly agree/agree/neutral/disagree/strongly disagree	57.2 (SD nor reported)	NR	NR	Neutral
Zinn et al. [51], 2006, New Zealand	Elite Coaches	Rugby	*n* = 364 (M)	NR	Existing Questionnaire; *n* = 88	Agree/Disagree/Unsure, Multi-Choice	55.6 (SD for total score not reported)	Supplements and performance (79.9 ± 18.9)	Micronutrients and Macronutrients (58.0 ± 19.4), Weight management and Eating disorders (63.8 ± 20.9), Hydration (61.9 ± 22.4)	Neutral

F = female; M = male; * = more than 1 point awarded for correct answers; NR = not reported; GNQK = general nutrition knowledge questionnaire; Bold = percent score calculated by researchers as either (a) scores presented as figure out of total (b) nutrition sub-section means but not total score reported; NA = not applicable; “Quality ratings were decided using the Academy of Nutrition and Dietetics” “Quality Criteria Checklist for Primary Research”.

**Table 3:** Comprehensiveness rating (score either 0 or 1 for each category).

Author	Items	General	CHO	Protein	Fat	Micro	Pre	During	Recovery	Fluid	Supplements	ETOH	Score
Abood et al. [32]	42	1	1	1	1	1	0	0	0	0	0	0	5
Alaunyte et al. [12]	28	1	1	1	1	1	0	0	0	0	0	0	4
Arazi and Hosseini [15]	40	1	1	1	1	1	U	U	U	U	U	0	4
Azizi et al. [46]	15	1	U	U	U	1	U	U	U	U	U	U	1
Barbaros-Tudor et al. [43]	87	1	1	1	1	1	1	0	0	1	1	0	7
Barr [48]	40	0	1	U	U	1	1	1	1	1	0	0	6
Botsis and Holden [44]	88	1	1	1	1	1	1	0	1	1	1	0	9
Collison et al. [34]	35	1	1	1	1	1	0	0	0	1	1	0	7
Condon et al. [35]	7	0	1	0	0	0	1	0	0	0	0	0	2
Corley et al. [53]	15	0	1	1	1	1	1	0	0	1	1	0	7
Danaher and Curley, 2014 [45]	88	1	1	1	1	1	1	0	1	1	1	0	9
Davar [17]	61	1	1	1	1	1	0	0	0	1	1	0	7
Devlin and Belski [16]	123	1	1	1	1	1	1	0	1	1	1	1	10
Dunn et al. [13]	124	1	1	1	1	1	0	0	0	0	0	0	5
Folasire et al. [18]	14	1	1	0	0	1	1	0	0	1	0	1	6
Grete R et al. [19]	20	1	1	1	1	1	1	0	0	1	0	0	7
Hamilton et al. [14]	48	1	1	1	1	1	1	0	0	1	1	0	8
Harrison et al. [47]	18	1	1	1	1	1	1	0	0	1	1	0	8
Hoogenboom et al. [52]	76	1	1	1	1	1	U	U	U	1	1	1	8
Jessri et al. [20]	88	1	1	1	1	1	1	0	1	1	1	0	9
Kunkel et al. [33]	31	1	1	1	U	U	U	U	U	U	U	U	3
Nichols et al. [42]	17	0	0	0	0	0	0	0	0	1	0	0	1
Rash et al. [36]	76	1	1	1	1	1	U	U	U	1	1	0	7
Raymond-Barker et al. [49]	110	1	1	1	1	1	0	0	0	0	0	0	5
Rosenbloom et al. [37]	11	0	1	1	1	0	1	0	0	1	1	0	6
Sedek and Yih [21]	29	1	1	1	1	1	1	0	0	1	1	1	9
Shifflett et al. [24]	20	1	1	1	1	1	0	0	1	1	1	0	8
Shoaf et al. [25]	25	1	1	1	1	1	1	0	0	0	1	0	7
Smith-Rockwell et al. [38]		1	1	1	1	1	0	0	0	1	1	0	7
Spendlove et al. [22]	113/90	1	1	1	1	1	0	0	0	0	0	0	5
Torres-McGehee et al. [23]	20	1	1	1	1	1	1	0	0	1	1	0	8
Weeden et al. [39]	24	1	1	1	1	1	0	0	0	1	1	1	8
Werblow et al. [40]	31	1	1	1	0	0	0	0	0	0	0	0	3
Worme et al. [50]	20	1	1	0	1	1	U	U	U	1	0	0	5
Zawila et al. [41]	76	1	1	1	1	1	U	U	U	1	1	1	8
Zinn et al. [51]	88	1	1	1	1	1	1	0	1	1	1	0	9

1 = adequate coverage of nutrition sub-section; 0 = inadequate coverage of nutrition sub-section; U = unclear (scored as 0). Decisions on whether a questionnaire included adequate coverage on each topic were made based on a combination of review of the actual tool (when available) and the description of the measure provided in the article.

**Table 4:** Validity and reliability rating score (either 0 or 1 for each category).

Author	Pre-Tested/Piloted	Face Validity	Content Validity	Item Discrimination	Internal Reliability	Construct Validity (Known Group Comparisons)	External Reliability	Total Score
Abood et al. [32]	Y; *n* = 6	0	1	0	0	0	1 (*r* = 0.86)	2
Alaunyte et al. [12]	N	1	1	1 *	P (*r* = 0.7–0.97)	1 *	1 *	5
Arazi and Hosseini [15]	N	1	1	0	0	0	0	2 +
Azizi et al. [46]	Y; *n* = 30	0	1	0	1 (α = 0.85)	0	0	2
Barbaros-Tudor et al. [43]	Y; *n* = 34 for construct; *n* = 10 for face	1	1	0	1 (*r* =0.82)	1 (dietitians > undergrads)	0	4
Barr, [48]	N	0	1	0	0	0	0	1
Botsis and Holden [44]	N	0	1	0	0	1 (dietitians > other groups)	1 (*r* = 0.74–0.93)	2
Collison et al. [34]	Y; *n* = 19	0	1	0	0	0	1	2
Condon et al. [35]	N	0	0	0	0	0	0	0
Corley et al. [53]	Y; *n* = 22	U	0	0	1(α = 0.56)	0	0	1
Danaher and Curley [45]	Y; *n* = NR	1	1	0	0	1 (dietitians > other groups)	1 (*r* = 0.74–0.93)	4
Davar [17]	Y; *n* = 5	1	1	0	0	0	0	2 +
Devlin and Belski [16]	N	0	0	0	0	0	0	0
Dunn et al. [13]	N	1	1	1	P (*r* = 0.7–0.97)	1 (nutrition > business)	1 (*r* = 0.7)	5
Folasire et al. [18]	Y	U	1	0	1 (α = 0.75)	0	0	2
Grete R et al. [19]	Y; *n* = NR	1	1	0	0	0	0	2 +
Hamilton et al. [14]	Y; *n* = NR	1	1	0	0	0	0	
Harrison et al. [47]	Y; *n* =10	1	0	0	0	0	0	1
Hoogenboom et al. [52]	N	0	0	0	0	0	0	0
Jessri et al. [20]	N	1	1	0	0	0	0	2 +
Kunkel et al. [33]	N	1	1	0	P (*r* = 0.82)	1	0	3
Nichols et al. [42]	N	0	1	0	0	0	0	1
Rash et al. [36]	Y; *n* = 20	0	0	0	1 (α = 0.94–0.96)	0	0	1
Raymond-Barker et al. [49]	Y; *n* = 47	1	1	0	0	0	0	2 +
Rosenbloom et al. [37]	Y; *n* = 6	1	1	1	P (*r* = 0.7–0.97)	P (nutrition > business)	1 (*r* = 0.7)	5
Sedek and Yih [21]	N	0	0	0	0	0	0	0
Shifflett et al. [24]	N	0	0	0	P (α = 0.645)	0	0	0
Shoaf et al. [25]	Y; *n* = 123	0	1	1	1 (not stated)	0	0	3
Smith-Rockwell et al. [38]	Y; *n* = 56	0	1	0	1 (α = 0.72)	0	1 (*r* = 0.82)	3
Spendlove et al. [22]	Y; *n* = 53	1	1	1 *	1 (α = 0.34–0.93 for GNKQ and 0.4–0.95 for R-GNKQ)	1 (nutrition > business)	1 (*r* = 0.37–0.92 in GNKQ)	6
Torres-McGehee et al. [23]	Y; *n* = 12	0	1	0	0	0	0	2
Weeden et al. [39]	Y; *n* = 21	1	1	0	0	0	0	2 +
Werblow et al. [40]	Y; *n* = 14	1	1	0	0	0	0	2 +
Worme et al. [50]	Y; *n* = NR	0	1	0	0	0	0	1
Zawila et al. [41]	N	0	0	1	0	0	0	1
Zinn et al. [51]	N	0	0	0	0	0	0	0
Abood et al. [32]	Y; *n* = 100	0	1	0	0	1*	1 * (*r* = 0.74–0.93)	3

Y = yes; N = no; *n* = number of participants; NR = 0 = psychometric validation not performed; 1 = psychometric validation performed; U = unclear; P = partial (internal consistency performed on original sample but not repeated), scored as 0; + = score of two, with both types of validation being qualitative (face and content validity but no quantitative statistical test performed); * = performed in original validation study but not-repeated in present study sample; *r* = Pearson’s or Spearman’s correlation coefficient as reported in paper (range represents scores across different nutrition sub-sections); α = Cronbach alpha value as reported in paper; NR = not reported.

## References

[1] Rodriguez N.R., DiMarco N.M., Langley S. (2009). Nutrition and athletic performance. Med. Sci. Sports Exerc..

[2] Potgieter S. (2013). Sport nutrition: A review of the latest guidelines for exercise and sport nutrition from the American College of Sport Nutrition, the International Olympic Committee and the International Society for Sports Nutrition. S. Afr. J. Clin. Nutr..

[3] Broad E.M., Cox G.R. (2008). What is the optimal composition of an athlete’s diet?. Eur. J. Sport Sci..

[4] Jonnalagadda S.S., Ziegler P.J., Nelson J.A. (2004). Food preferences, dieting behaviors, and body image perceptions of elite figure skaters. Int. J. Sport Nutr. Exerc. Metab..

[5] Sawka M.N., Burke L.M., Eichner E.R., Maughan R.J., Montain S.J., Stachenfeld N.S. (2007). American College of Sports Medicine position stand. Exercise and fluid replacement. Med. Sci. Sports Exerc..

[6] Spronk I., Kullen C., Burdon C., O’Connor H. (2014). Relationship between nutrition knowledge and dietary intake. Br. J. Nutr..

[7] Heaney S., O’Connor H., Michael S., Gifford J., Naughton G. (2011). Nutrition knowledge in athletes: A systematic review. Int. J. Sport Nutr. Exerc. Metab..

[8] Heaney S., O’Connor H., Naughton G., Gifford J. (2008). Towards an understanding of the barriers to good nutrition for elite athletes. Int. J. Sports Sci. Coach..

[9] Ono M., Kennedy E., Reeves S., Cronin L. (2012). Nutrition and culture in professional football. A mixed method approach. Appetite.

[10] Birkenhead K.L., Slater G. (2015). A review of factors influencing athletes’ food choices. Sports Med..

[11] Clark K.S. (1999). Sports nutrition counseling: Documentation of performance. Top. Clin. Nutr..

[12] Alaunyte I., Perry J.L., Aubrey T. (2015). Nutritional knowledge and eating habits of professional rugby league players: Does knowledge translate into practice?. J. Int. Soc. Sports Nutr..

[13] Dunn D., Turner L.W., Denny G. (2007). Nutrition knowledge and attitudes of college athletes. Sport J..

[14] Hamilton G., Thomson C., Hopkins W. (1994). Nutrition knowledge of elite distance runners. N. Z. J. Sports Med..

[15] Arazi H., Hosseini R. (2012). A comparison of nutritional knowledge and food habits of collegiate and non-collegiate athletes. SportLogia.

[16] Devlin B.L., Belski R. (2015). Exploring general and sports nutrition and food knowledge in elite male Australian athletes. Int. J. Sport Nutr. Exerc. Metab..

[17] Davar V. (2012). Nutritional knowledge and attitudes towards healthy eating of college-going women hockey players. J. Hum. Ecol..

[18] Folasire O.F., Akomolafe A.A., Sanusi R.A. (2015). Does nutrition knowledge and practice of athletes translate to enhanced athletic performance? Cross-sectional study amongst nigerian undergraduate athletes. Glob. J. Health Sci..

[19] Grete R.H., Carol A.F., Jane E.E., Kimberli P. (2011). Nutrition knowledge, practices, attitudes, and information sources of mid-American conference college softball players. Food Nutr. Sci..

[20] Jessri M., Jessri M., RashidKhani B., Zinn C. (2010). Evaluation of Iranian college athletes’ sport nutrition knowledge. Int. J. Sport Nutr. Exerc. Metab..

[21] Sedek R., Yih T.Y. (2014). Dietary habits and nutrition knowledge among athletes and non-athletes in National University of Malaysia (UKM). Pak. J. Nutr..

[22] Spendlove J.K., Heaney S.E., Gifford J.A., Prvan T., Denyer G.S., O’Connor H.T. (2012). Evaluation of general nutrition knowledge in elite Australian athletes. Br. J. Nutr..

[23] Torres-McGehee T.M., Pritchett K.L., Zippel D., Minton D.M., Cellamare A., Sibilia M. (2012). Sports nutrition knowledge among collegiate athletes, coaches, athletic trainers, and strength and conditioning specialists. J. Athl. Train..

[24] Shifflett B., Timm C., Kahanov L. (2002). Understanding of athletes’ nutritional needs among athletes, coaches, and athletic trainers. Res. Q. Exerc. Sport.

[25] Shoaf L.R., McClellan P.D., Birskovich K.A. (1986). Nutrition knowledge, interests, and information sources of male athletes. J. Nutr. Educ..

[26] International Prospective Register of Systematic Reviews. http://www.crd.york.ac.uk/PROSPERO/.

[27] Parmenter K., Wardle J. (2000). Evaluation and design of nutrition knowledge measures. J. Nutr. Educ..

[28] Kline P. (2013). Handbook of Psychological Testing.

[29] Nunnally J. (1978). Psychometric Methods.

[30] Streiner D.L. (2003). Starting at the beginning: An introduction to coefficient alpha and internal consistency. J. Personal. Assess..

[31] American Dietetic Association Evidence Analysis Manual: Steps in the Ada Evidence Analysis. https://www.adaevidencelibrary.com/vault/editor/File/Evidence_Analysis_Manual_January_2008.pdf.

[32] Abood D.A., Black D.R., Birnbaum R.D. (2004). Nutrition education intervention for college female athletes. J. Nutr. Educ. Behav..

[33] Kunkel M.E., Bell L.B., Luccia B.H.D. (2001). Peer nutrition education program to improve nutrition knowledge of female collegiate athletes. J. Nutr. Educ..

[34] Collison S.B., Kuczmarski M.F., Vickery C.E. (1996). Impact of nutrition education on female athletes. Am. J. Health Behav..

[35] Condon E.M., Dube K.A., Herbold N.H. (2007). The influence of the low-carbohydrate trend on collegiate athletes’ knowledge, attitudes, and dietary intake of carbohydrates. Top. Clin. Nutr..

[36] Rash C.L., Malinauskas B.M., Duffrin M.W., Barber-Heidal K., Overton R.F. (2008). Nutrition-related knowledge, attitude, and dietary intake of college track athletes. Sport J..

[37] Rosenbloom C.A., Jonnalagadda S.S., Skinner R. (2002). Nutrition knowledge of collegiate athletes in a Division I National Collegiate Athletic Association institution. J. Am. Diet. Assoc..

[38] Smith-Rockwell M., Nickols-Richardson S.M., Thye F.W. (2001). Nutrition knowledge, opinions, and practices of coaches and athletic trainers at a division 1 university. Int. J. Sport Nutr. Exerc. Metab..

[39] Weeden A., Olsen J., Batacan J., Peterson T. (2014). Differences in collegiate athlete nutrition knowledge as determined by athlete characteristics. Sport J..

[40] Werblow J.A., Fox H.M., Henneman A. (1978). Nutritional knowledge, attitudes, and food patterns of women athletes. J. Am. Diet. Assoc..

[41] Zawila L.G., Steib C.M., Hoogenboom B. (2003). The female collegiate cross-country runner: Nutritional knowledge and attitudes. J. Athl. Train..

[42] Nichols P.E., Jonnalagadda S.S., Rosenbloom C.A., Trinkaus M. (2005). Knowledge, attitudes, and behaviors regarding hydration and fluid replacement of collegiate athletes. Int. J. Sport Nutr. Exerc. Metab..

[43] Barbaros-Tudor P., Radman I., Jankovic G. Nutritional knowledge and dietary habits of croatian tennis coaches. Proceedings of the 6th International Scientific Conference on Kinesiology: Integrative Power of Kinesiology.

[44] Botsis A.E., Holden S.L. (2015). Nutritional knowledge of college coaches. Sport Sci. Rev..

[45] Danaher K., Curley T. (2014). Nutrition knowledge and practices of varsity coaches at a Canadian university. Can. J. Diet. Pract. Res..

[46] Azizi M., Rahmani-Nia F., Malaee M., Malaee M., Khosravi N. (2010). A study of nutritional knowledge and attitudes of elite college athletes in Iran. Braz. J. Biomot..

[47] Harrison J., Hopkins W., MacFarlane D., Worsley A. (1991). Nutrition knowledge and dietary habits of elite and non-elite athletes. Aust. J. Nutr. Diet..

[48] Barr S. (1987). Nutrition knowledge of female varsity athletes and university students. J. Am. Diet. Assoc..

[49] Raymond-Barker P., Petroczi A., Quested E. (2007). Assessment of nutritional knowledge in female athletes susceptible to the Female Athlete Triad syndrome. J. Occup. Med. Toxicol..

[50] Worme J.D., Doubt T.J., Singh A., Ryan C.J., Moses F.M., Deuster P.A. (1990). Dietary patterns, gastrointestinal complaints, and nutrition knowledge of recreational triathletes. Am. J. Clin. Nutr..

[51] Zinn C., Schofield G., Wall C. (2006). Evaluation of sports nutrition knowledge of New Zealand premier club rugby coaches. Int. J. Sport Nutr. Exerc. Metab..

[52] Hoogenboom B.J., Morris J., Morris C., Schaefer K. (2009). Nutritional knowledge and eating behaviors of female, collegiate swimmers. N. Am. J. Sports Phys. Ther..

[53] Corley G., Demarest-Litchford M., Bazzarre T.L. (1990). Nutrition knowledge and dietary practices of college coaches. J. Am. Diet. Assoc..

[54] Zinn C., Schofield G., Wall C. (2005). Development of a psychometrically valid and reliable sports nutrition knowledge questionnaire. J. Sci. Med. Sport.

[55] Parmenter K., Wardle J. (1999). Development of a general nutrition knowledge questionnaire for adults. Eur. J. Clin. Nutr..

[56] Spronk I., Heaney S.E., Prvan T., O’Connor H.T. (2015). Relationship between general nutrition knowledge and dietary quality in elite athletes. Int. J. Sport Nutr. Exerc. Metab..

[57] O’Brien K.S., Kypri K. (2008). Alcohol industry sponsorship and hazardous drinking among sportspeople. Addiction.

[58] Martens M.P., Dams-O’Connor K., Beck N.C. (2006). A systematic review of college student-athlete drinking: Prevalence rates, sport-related factors, and interventions. J. Subst. Abuse Treat..

[59] Maughan R., Greenhaff P., Hespel P. (2011). Dietary supplements for athletes: Emerging trends and recurring themes. J. Sports Sci..

[60] Goulet E.D. (2011). Effect of exercise-induced dehydration on time-trial exercise performance: A meta-analysis. Br. J. Sports Med..

[61] Beaton D.E., Bombardier C., Guillemin F., Ferraz M.B. (2000). Guidelines for the process of cross-cultural adaptation of self-report measures. Spine.

[62] Paugh S.L. Dietary Habits And Nutritional Knowledge of College Athletes. http://libweb.calu.edu/thesis/umi-cup-1011.pdf.

[63] Thompson B. (2004). Exploratory and Confirmatory Factor Analysis: Understanding Concepts and Applications.

[64] Tennant A., Conaghan P.G. (2007). The Rasch measurement model in rheumatology: What is it and why use it? When should it be applied, and what should one look for in a Rasch paper?. Arthritis Care Res..

